# Transdermal Drug Delivery: Determining Permeation Parameters Using Tape Stripping and Numerical Modeling

**DOI:** 10.3390/pharmaceutics14091880

**Published:** 2022-09-06

**Authors:** Fjola Jonsdottir, Bergthora S. Snorradottir, Skuli Gunnarsson, Elina Georgsdottir, Sven Sigurdsson

**Affiliations:** 1 Faculty of Industrial Engineering, Mechanical Engineering and Computer Science, University of Iceland, 107 Reykjavik, Iceland; 2 Faculty of Pharmaceutical Sciences, University of Iceland, 107 Reykjavik, Iceland

**Keywords:** transdermal, diffusion, numerical model, partition, mass transfer, tape stripping

## Abstract

The function of transdermal drug delivery (TDD) systems is complex due to the multiple layers necessary for controlling the rate of drug release and the interaction with the patient’s skin. In this work, we study a particular aspect of a TDD system, that is, the parameters that describe the drug permeation through the skin layers. Studies of the diffusion of two compounds were carried out and supported by tape stripping and numerical modeling. The experimental studies are carried out for porcine skin in a Franz diffusion cell and tape stripping is used to quantify the concentration of drug in the stratum corneum. A multi-layered numerical model, based on Fickian diffusion, is used to determine the unknown parameters that define the skin’s permeability, such as the partition between layers and the mass transfer coefficients due to the surface barrier. A significant correlation was found between the numerical modeling and experimental results, indicating that the partition and mass transfer effects at the interlayer boundary are accurately represented in the numerical model. We find that numerical modeling is essential to fully describe the diffusion characteristics.

## 1. Introduction

Transdermal drug delivery (TDD) systems, such as patches are increasingly replacing traditional drug delivery methods [1]. Examples of drugs administered through transdermal patches include scopolamine for motion sickness, nicotine for smoking cessation aid, estrogen for menopause and to prevent osteoporosis after menopause, rivastigmine or acetylcholine inhibitor, used for the improvement of behavioral, and psychological symptoms of dementia [2,3,4,5]. The function of TDD systems is complex due to the multiple layers necessary for controlling the rate of drug release and the interaction of the patch with the patient’s skin. Furthermore, the skin layer itself is composed of multiple layers, each with different permeability qualities. There are three main layers of skin: epidermis, living dermis, and subcutaneous tissues or hypodermis. Each of these layers performs a significant role in protecting the body and maintaining overall health [6,7,8].

Skin, the largest and most easily accessible organ of the human body, is an attractive target for therapeutic applications over other drug administration routes, offering a safe, convenient and painless way for drug administration. Topical application of drugs provides a relatively constant drug-release rate over long periods of time; it enables instant termination of drug input, minimizes the risk of undesirable side effects, etc., [9,10,11]. However, topical and TDD systems do have some disadvantages. The low permeability of the skin, due in large part to the Stratum Corneum (SC), the outermost layer of the epidermis, remains the greatest challenge in the delivery of topically applied active ingredients. Effective skin permeation is therefore limited to small, lipophilic, potent molecules with a relatively low melting point (<200 °C) [3,12,13,14,15].

Tape stripping (TS) is a widely used method in transdermal drug delivery to quantify the drug that is retained in the SC at the end of an experiment [16]. Thus, it can be used to determine the partition coefficient between the donor compartment and the skin by finding the ratio of drug concentration between the two [17]. The TS technique is minimally invasive where the SC’s cell layers are peeled from the same skin area, using sticky sheets after topical application and penetration of formulations [16,18].

Mathematical modeling has been used in the design of drug delivery systems for over 50 years [19]. Mathematical models for TDD are generally based on Fickian diffusion and solved numerically for given boundary conditions [20,21,22,23]. Important aspects of such models include the appropriate interface conditions to account for partition effects and possible surface barriers that lead to discontinuities in drug concentration across interfaces.

In this study, we focus on the diffusion of the drug through the skin layers and the quantification of the drug in different layers of the skin, using the tape stripping technique and numerical modeling. The nonsteroidal anti-inflammatory drug diclofenac and the chemical compound caffeine are chosen for the study. Permeation studies are carried out for porcine skin in a Franz diffusion cell and tape stripping is used to measure the effectiveness of the local drug permeation. A multi-layered numerical model is developed, based on Fickian diffusion, which accounts for both partitions between the system’s layers and the mass transfer effects, due to the surface barriers. The model is an extension of the numerical model employed by Gudnason et al. [24], based on additional and more accurate experimental results achieved using the tape stripping technique.

## 2. Materials and Methods

### 2.1. Materials

Acetic acid, diclofenac sodium salt, caffeine, and sodium chloride were purchased from Sigma-Aldrich. Acetonitrile (HPLC grade), methanol (HPLC grade), tetrahydrofuran (HPLC grade), and potassium phosphate monobasic were purchased from Riedel-de Haen.

### 2.2. HPLC Analysis

HPLC quantification was carried out using an Ultimate 3000 from Dionex, methods settings are presented in Table 1. The column used was 150 × 4.6 mm, 5 µm, C-8 from YMC.

### 2.3. Release Studies by In Vitro Experiments

Due to ethical reasons, porcine ear skin was chosen for the experimentation. Porcine skin is considered to be the most human-relevant animal model used for dermal/transdermal research [25,26,27]. Porcine ears were obtained from freshly killed animals (6-month-old) from a local abattoir Stjörnugrís (since the ear is often a by-product in their production). During the transportation, the ears were stored in ice. Immediately after receipt in the lab, the ears were washed with cold distilled water and dried using soft tissue. Skin was further prepared by removing the whole skin carefully from the underlying cartilage. The initial thickness of the skin membranes was 1.6 mm ± 2 mm. Skin membranes were stored on aluminum foil at −20 °C until used. Just before the experiment began, skin samples were thawed at room temperature and dermatomed to nominal thickness (ca. ≥ 1 mm), using the manual dermatome or sterile scalpel blades [28]. The experimentally obtained thickness was determined using a digital caliper. All prepared skin samples were punched to 1.5 cm disks and analyzed using optical microscopy in order to exclude damaged skin samples.

**Figure 1:**
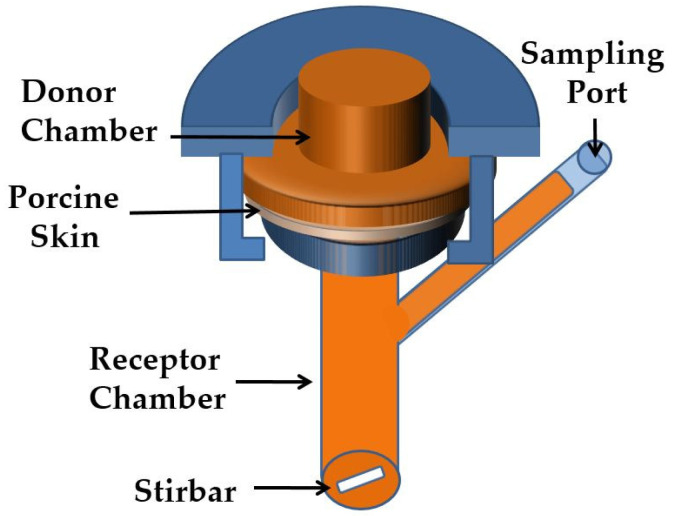
Schematic illustration of unjacketed Franz diffusion cell.

In vitro percutaneous permeation studies were carried out using unjacketed Franz diffusion cells; see Figure 1, with an orifice diameter of 0.9 cm (area of exposure 0.64 cm^2^), a donor chamber volume of 1 mL, and a receptor chamber volume of 12 mL.

PBS was prepared accordingly to the guidance of European Pharmacopoeia, from buffer solution preparations [29]. Dermatomed skin membrane discs were immersed in PBS until further processing, four replicates per experiment. Degassed PBS (pH 7.4) was used as the receptor phase. Subsequently, pre-prepared discs were carefully placed at the interface between the donor and receptor compartments so that the epidermal side was facing the donor compartment. Franz diffusion cells were mounted on a magnetic stirrer plate at 400 rpm [11] and equilibrated for 30 min. The diffusion cells remained in an oven at a constant temperature of 32 ± 1 °C. At time zero, 1 mL of test substances of known concentration 5 mg/mL, were applied directly into the donor compartment and sealed with paraffin film to prevent evaporation of the solutions. At predetermined time intervals, receptor fluid was sampled via sampling arm, 200 µL each time, using a disposable syringe. Samples were taken at pre-determined time intervals: 1, 2, 3, 4.5, 6, 8, 10, 12, 23, 26, 29, 32, 35 and 48 h. Removed volume was replaced with an equivalent volume of fresh receptor fluid. Samples were analyzed by HPLC.

### 2.4. Tape Stripping In Vitro

Following the sampling period, each skin sample was removed from the Franz cell and rinsed with 2 mL of PBS. The sample of the skin surface was dried with cotton wool prior to tape stripping. The adhesive tape strips were prepared in advance. Further, the SC was removed by employing the TS method. Application of the adhesive tape was followed by uniform, gentle pressure (2 kg) rolling the tape twice onto the skin surface. SC was sequentially removed from the same skin area by repeated tape strip application and taken off by sharp upward movement. Tape strip removal was performed with relatively constant velocity. Each skin sample was stripped 70 times. The thickness of each tape strip is 0.5–1 µm, and hence 70 strips stripped away a total thickness of 0.0035–0.0070 cm. The tape strips, as well as the rest of the skin and subcutaneous fat, were placed into the 1.5 mL microtubes (2 tapes per tube, rest of the skin and subcutaneous fat placed separately), with 1 mL of methanol. Samples were sonicated for 15 min for extraction and then centrifuged for 10 min at 10,000 rpm. Extract aliquots were analyzed and quantified by HPLC.

### 2.5. Numerical Simulations

The transportation of drugs through the skin may be described by the Fickian diffusion equation, where the key parameter is the diffusion coefficient. Other key parameters that define the skin permeability are the partition coefficient, due to the difference in solubility between layers, and the mass transfer coefficient, due to surface barriers between layers. Both parameters lead to a discontinuous jump in concentration at the interlayer boundary [30].

**Figure 2:**
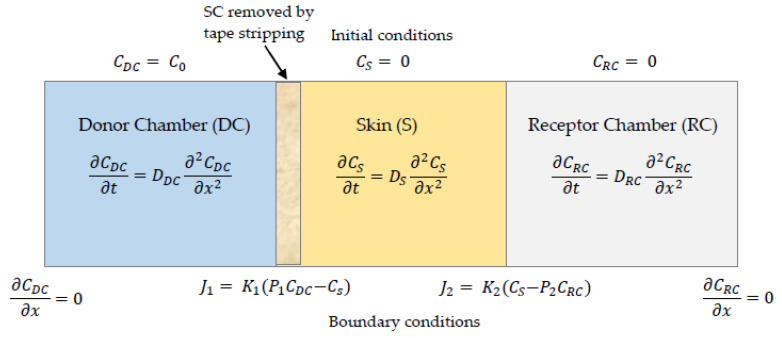
Schematic of the numerical model showing initial and boundary conditions.

The mathematical model simulates a Franz diffusion cell system. A one-dimensional, three-layer model was created, comprised of the donor chamber (DC), the skin, and the receptor chamber (RC), as shown in Figure 2. The drug is loaded in the DC, which can represent a patch, then travels through the skin and enters the RC. Within each layer *i*, where *i* = (1,2,3) for a three-layer model, the diffusion in the x-direction is governed by Fick´s equation:

(1) $$\frac{\partial C_{i}\left(x,t\right)}{\partial t}=\frac{\partial }{\partial x}\left(D_{i}\frac{\partial C_{i}\left(x,t\right)}{\partial x}\right)$$

where

$C_{i}\left(x,t\right)$ is the concentration of dissolved drug within *i*-th layer (mg/cm^3^) and

$D_{i}$ is the diffusion coefficient of dissolved drug within *i*-th layer (cm^2^/h).

Within each layer, the initial condition is:

(2) $$C_{i}\left(x,0\right)=C_{i,0}.$$

Interlayer boundary conditions are defined between layers that describe the combined mechanisms of partition and mass transfer rate. The interlayer conditions for *i* = [1,2] are:

(3) $$J_{i}=-D_{i}{\frac{\partial C_{i}\left(x,t\right)}{\partial x}|}_{x=x_{i}-}=-D_{i+1}{\frac{\partial C_{i+1}\left(x,t\right)}{\partial x}|}_{x=x_{i}+}=K_{i}\left(C_{i}\left(x_{i},t\right)-P_{i}C_{i+1}\left(x_{i},t\right)\right)$$

where

$K_{i}$ is the mass transfer coefficient of dissolved drug between layers  i  and i+1 (cm/h),

$P_{i}$ is the partition coefficient of dissolved drug between layers i and i+1 (-) and

$J_{i}$ is the flux between layers i and i+1 (mg/h-cm^2^).

The value of $P_{i}\;$ gives the ratio between concentrations on each side of the boundary at equilibrium and $K_{i}$ is a rate coefficient, indicating how quickly equilibrium is reached.

A high $K_{i}$ value means that there is no barrier at the boundary, whereas a small value indicates the presence of a barrier.

The mathematical model is solved numerically with the finite element method, incorporating the boundary conditions. An implicit Euler method is used for the time integrations. For further details on the numerical model see Gudnason et al. [21].

The unknown partition and mass transfer coefficients are obtained such that the simulated release curve in the RC matches the experimental results, and furthermore, that the concentration of drug in the SC layer of the skin matches the tape stripping results. That is, P_1_ is estimated from the ratio of the concentration in the SC layer of the skin and the concentration in the DC. After obtaining a value for P_1_, the other parameters P_2_, K_1_, and K_2_ are obtained by matching the RC experimental results as closely as possible. The diffusion coefficient in the skin is determined experimentally by using the lag time method [31]. However, the diffusion coefficients in the DC and the RC are kept large enough for uniform distribution of the drug.

## 3. Results

Experiments and numerical simulations were carried out for three cases: diclofenac at room temperature (RT), diclofenac at 32 °C, and caffeine at 32 °C. For each case, the results from tape stripping and permeation studies are shown, along with simulated release curves and concentration profiles in the skin at different times. The estimated permeation parameter values are given for each case. All experimental results are shown as mean values of all repetitions, with error bars indicating standard deviation.

### 3.1. Case 1: Diclofenac Solution at Room Temperature

**Figure 3:**
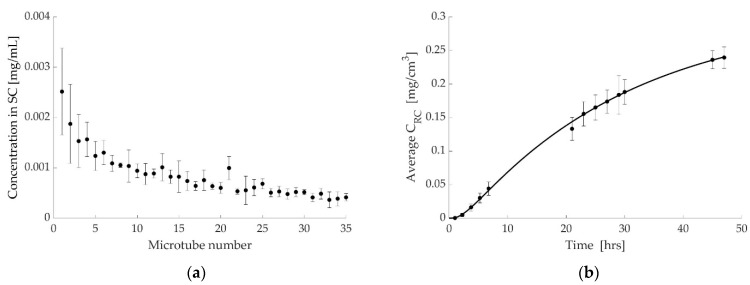

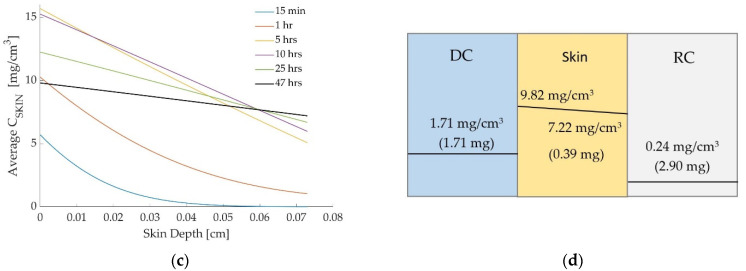
Diclofenac at room temperature. (**a**) Tape stripping results (mean of all replicates ± standard deviation (*n* = 4)). Microtube no. 1 represents tapes no. 1–2 in the top layer of SC and no. 35 represents tapes no. 69–70. (**b**) Simulated release curve (solid line) and experimental results (marker). (**c**) Simulated concentration profiles in the skin at different times. (**d**) The concentration and the amount of drug in each layer at the end of the simulation, showing discontinuity between layers.

The drug concentrations in the SC, generated from the in vitro skin tape stripping experiments, are shown in Figure 3a. By adding the amount of drug in all strips, the total mean value of drug retained in the strips is estimated to be 0.032 mg. The experimental results for drug concentration in the RC (marked with circles) are shown in Figure 3b, along with the simulated release curve. The simulated curve fits all experimental data points accurately, except for one point which is still within the margin of error. Figure 3d demonstrates how the drug concentration changes with time through the different layers of the skin. As the experiment was run for 47 h the simulation is also run for 47 h. To obtain P_1_, we match the amount of drug in the simulation with the amount from the tape stripping experiment. As each tape strip is 0.5 µm to 1 µm thick, the total thickness for 70 strips is 0.0035 cm to 0.0070 cm. Hence, to obtain the amount of drug, we compute the area under the 47-h line (black line) in Figure 3c, from 0 to 0.0035 cm and from 0 to 0.0070 cm, and multiply by the area of the membrane (0.636 cm^2^). Carrying out these calculations, we get a total amount of drug of about 0.022 mg at a depth of 0.0035 cm and about 0.043 mg at a depth of 0.0070 cm. The average of these two numbers gives 0.032 mg which matches the experimental result obtained from Figure 3a.

After estimating P_1_, the rest of the parameters, P_2_, K_1_, and K_2_, are obtained by simulating the RC release curve. The estimated parameter values are given in Table 2, along with a summary of data used in the experiments and simulations.

As the drug increases in the RC, it decreases in the DC and the amount is calculated by the numerical model at each time. Figure 3d shows the discontinuity between layers schematically and gives the drug concentration in the DC at the end of the simulation (47 h), along with concentration values for the skin and the RC (same values as in Figure 3b,c) and the amount of drug in each layer.

### 3.2. Case 2: Dicofenac Solution at 32 °C

**Figure 4:**
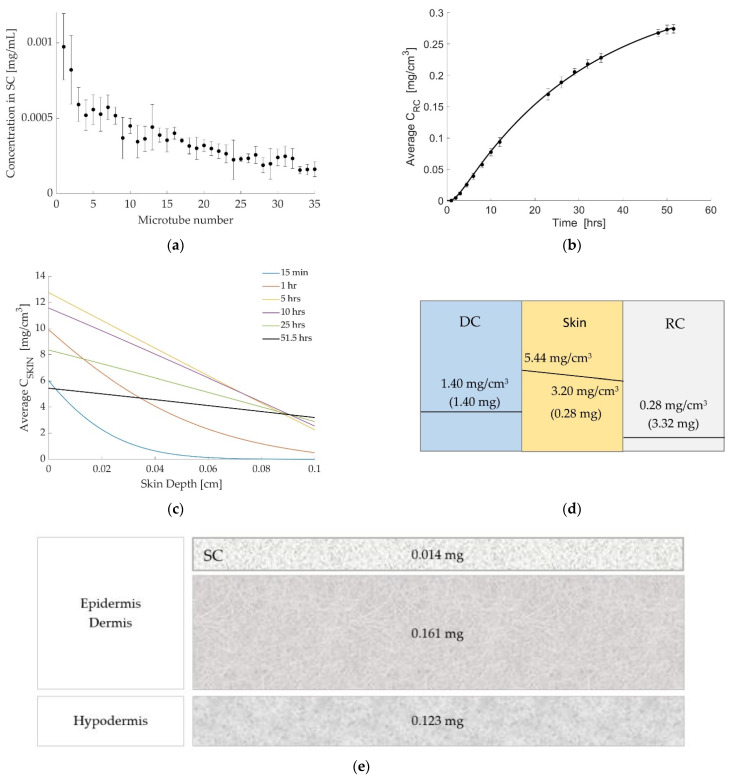
Diclofenac at 32 °C. (**a**) Tape stripping results (mean of all replicates ± standard deviation). Microtube no. 1 represents tapes no. 1–2 in the top layer of SC and no. 35 represents tapes no. 69–70. (**b**) Simulated release curve (solid line) and experimental results (marker). (**c**) Simulated concentration profiles in the skin at different times. (**d**) The concentration and the amount of drug in each layer at the end of the simulation, showing discontinuity between layers. (**e**) The measured amount of drug in the stratum corneum, rest of epidermis and dermis, and hypodermis at the end of the experiment.

The concentration profiles generated from the in vitro skin tape stripping experiments are shown in Figure 4a. Again, by adding up the magnitude in all the strips, the mean total value of the drug retained in the strips is 0.014 mg. The experimental results for drug concentration in the RC (marked with circles) are shown in Figure 4b, along with the simulated release curve. Again, the simulated results fit well with experimental data.

Figure 4c shows the simulated drug concentration profiles in the skin at different time values. This experiment ran for 51.5 h and hence we run the simulation for 51.5 h. We use the same method as explained above to estimate the value of P_1_. In this case, the simulated amount of drug is between 0.012 mg (at a depth of 0.0035 cm) and 0.024 mg (at a depth of 0.0070 cm) which matches well with the experimental result of 0.014 mg. All the estimated values of partition coefficients and mass transfer coefficients are given in Table 3, along with a summary of data used in the experiments and simulations. Figure 4d gives the drug concentration and the amount of drug in each layer at the end of the simulation and shows the discontinuity between layers schematically. Furthermore, for this case, we measured the amount of drug retained in the SC, the rest of the epidermis and dermis, and hypodermis at the end of the experiment. The results are shown in Figure 4e and were found to closely match the computed amount of drug as shown in Figure 4d.

### 3.3. Case 3: Caffeine at 32 °C

**Figure 5:**
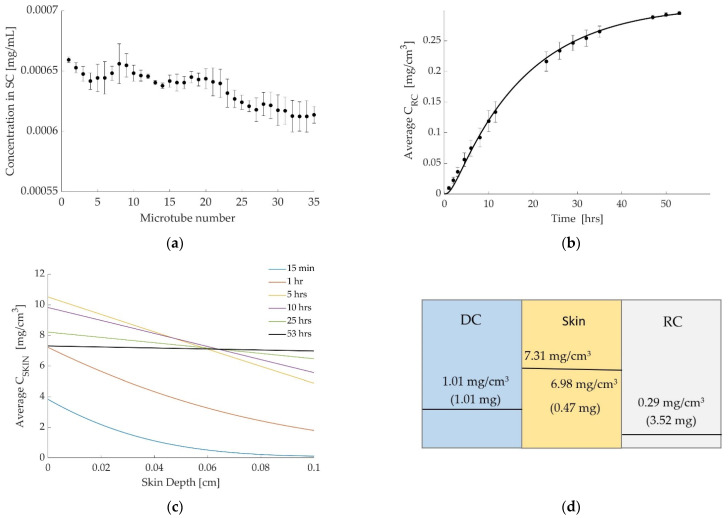
Caffeine at 32 °C. (**a**) Tape stripping results (mean of all replicates ± standard deviation). Microtube no. 1 represents tapes no. 1–2 in the top layer of SC and no. 35 represents tapes no. 69–70. (**b**) Simulated release curve (solid line) and experimental results (marker). (**c**) Simulated concentration profiles in the skin at different times. (**d**) The concentration and the amount of drug in each layer at the end of the simulation, showing discontinuity between layers.

The concentration profiles generated from the in vitro skin tape stripping experiments are shown in Figure 5a. The mean value for the total amount of drug retained in the strips is found to be 0.022 mg.

Figure 5b,c show the results from the permeation study (marked with circles), along with the simulated release curve, and the simulated drug concentration profiles, respectively. The same method, as explained previously, is used for estimating the value for P_1_. In this case, the simulated amount of drug is between 0.016 mg (at a depth of 0.0035 cm) and 0.032 mg (at a depth of 0.0070 cm), which matches well with the experimental result of 0.022 mg.

The values estimated for partition coefficients and mass transfer coefficients are given in Table 4, along with a summary of data used in the experiments and simulations. Figure 5d gives the drug concentration and the magnitude of the drug in each layer at the end of the simulation and shows the discontinuity between layers schematically.

## 4. Discussion

The transdermal studies were carried out for approximately 48 h, as full-thickness porcine skin was used. The integrity of the skin may start to deteriorate after 24 h [25]. For the full thickness, porcine skin used preliminary studies suggested that the skin would be expected to maintain integrity for up to 48 h. The integrity was assessed by visual examination via microscope.

There are a number of factors that affect transdermal drug delivery. The degree of ionization of the drug molecule at a particular pH is very important for the extent of penetration, and unionized molecules pass barriers of the skin much better than those that are ionic [12,32,33]. Partition coefficient (log P) is also considered to be an important and frequently used physicochemical property for predicting skin permeability. Therefore, both diclofenac and caffeine were chosen as model drugs. The current permeation results suggest that the physicochemical properties of the compounds are interconnected with their permeation through the skin in the current research. SC is considered to be the primary rate-limiting step to transdermal drug absorption. Therefore, the penetrant concentration in the outermost layer of the skin may apparently be related to the drug concentrations in deeper tissues. The drug amount retained in SC was identified using the TS technique. Sink conditions can affect the transdermal drug delivery and in this study, no additives were added to the receptor media to avoid any enhancing effect of such additives, removed samples were replaced with fresh PBS but alone cannot assure full sink condition. In this study not adding any excipients allows us to test the mathematical model and compare the two model drugs before we enhance our model to account for excipients.

As may be noticed in Figure 3a, Figure 4a and Figure 5a, the amount of penetrant decreases with increased depth into the skin. The reason for this may be due to increased cohesion of cells, which increases with SC depth. The TS results suggest that the physicochemical properties of the compounds are interconnected with the drug amounts retained in the SC. The given values are interconnected with those values obtained during Franz cell diffusion studies, e.g., a relationship with the diffusion coefficient of the drug was observed.

This study showed that skin permeation of caffeine and diclofenac at different temperatures were in proportion with their physicochemical properties, and the flux of the compounds increased with decreasing molecular mass and partition coefficient. It was observed that a lower partition coefficient showed better permeation, which can be related to the hydration of the skin. The data obtained during Franz diffusion was within the expected range.

Current TS results suggest that the physicochemical properties of the compounds are as well in proportion with the drug amounts retained in the SC. The amount of drug retained in the skin increased with decreasing partition coefficient or molecular mass. The data obtained during the TS experiment was within the expected range.

Drug amounts in the lower layers of the skin were examined as well. It was found that caffeine and diclofenac had a higher tendency to linger into the epidermis/dermis rather than the subcutaneous layer or fat.

The modeling results fit the experimental data closely, as shown in Figure 3b, Figure 4b and Figure 5b. The coefficients computed are shown in Table 2, Table 3 and Table 4 and demonstrate similar characteristics in all three cases.

As the results in Table 2, Table 3 and Table 4 show, both mass transfer coefficients, K_1_ and K_2_, have low values in all three cases. A low value for K_1_ demonstrates the resistance of the SC to the drug uptake, whereas a low value of K_2_ demonstrates the barrier at the boundary between the skin and the RC. The value of K determines the rate of transition from the initial stage to equilibrium. Hence, a high value of K means that equilibrium is reached almost instantaneously, whereas a low value of K means it takes longer for the system to reach equilibrium. The low K values cause the behavior shown in Figure 3c, Figure 4c and Figure 5c in which the concentration at the top of the skin increases up to a certain point before decreasing again as the system approaches equilibrium.

Looking at Figure 3c (case 1), for example. At a time of 15 min the calculated DC concentration is 4.94 mg/cm^3^ and if there were no barrier effect the concentration at the top of the skin should be 39.52 mg/cm^3^. The fact that it is only 5.72 mg/cm^3^ is due to the barrier effect causing a negative jump in concentration between DC and skin. However, this barrier effect decreases as time passes and we get closer to equilibrium, causing an increase in concentration at the top of the skin. However, this increase is counteracted by the decreasing concentration in DC which eventually starts to dominate at approximately 5 h. At 47 h, when we are approaching equilibrium, the calculated DC concentration is 1.71 mg/cm^3^ whereas the concentration at the top of the skin is 9.82 mg/cm^3^. The ratio of these two values is in agreement with the partition coefficient being P_1_ = 8.

The behavior caused by the low K values is also present at the boundary between the skin and the RC. Again, consider Figure 3c as an example. Early on, the concentration in the bottom layer of the skin is minuscule but increases with time. Looking at the discontinuity between the skin and the RC, we see that the values are greater than the value of P_2_ for all time values. Again, the low K value causes a negative jump in the RC value and hence adds to the discontinuity caused by the partition. Similar behavior is observed in the other two cases.

When equilibrium is reached, the difference in concentration in the layers is caused by the partition coefficient alone; the mass transfer coefficient has no effect at this stage. None of our cases have completely reached equilibrium, but the caffeine trials (case 3) are the one that is closest. This is shown by the almost flat line in Figure 5c for a time of 53 h. Saturation due to lack of sink condition is possible, but the mathematical model can adjust to that. Furthermore, Figure 5d shows that the ratio of the concentration values across the DC/skin boundary, that is, 7.31 mg/cm^3^/1.01 mg/cm^3^ approaches the value of P_1_ = 8, and the ratio of the concentration values across the skin/RC boundary, that is, 6.98 mg/cm^3^/0.29 mg/cm^3^ is close to the value of P_2_ = 23; see Table 4. It is noted that in all three cases studied, the values for P_2_ are higher than values for P_1_; see Table 2, Table 3 and Table 4. This means that there is a greater discontinuity between the RC and the skin than between the DC and the skin.

## 5. Conclusions

Drug permeation experiments using porcine skin in a Franz diffusion cell were carried out, supported by tape stripping and numerical modeling. The tape stripping method proved to be a valuable addition to the permeation experiments in this study and was used to quantify the drug in different layers of the skin, as well as determine the partition between layers. The numerical simulations were determined to be essential to fully characterize the diffusion characteristics of a layered system, such as porcine skin, accurately representing the combined partition and mass transfer effects at the interlayer boundaries. A close correlation was found between the experimental diffusion studies, tape stripping, and the numerical model, indicating that the characteristics are accurately represented by the mathematical modeling.

## Figures and Tables

**Table 1:** Method settings for HPLC analysis.

Settings	Mobile Phase	Flow Rate mL/min	UV Signal nm	Retention Time min
Diclofenac	Acetonitrile: 1% acetic acid (40:60)	1.5	281	2.2
Caffeine	Methanol: MilliQ water (40:60)	0.8	272	4.1

**Table 2:** Estimated parameter values for simulations of diclofenac at room temperature.

Starting Concentration C_0_	5.00 mg/mL
Experiment time	47 h
Skin thickness	0.07 cm
Diffusion coefficient	1.2 × 10^−3^ cm^2^/h
Partition coefficient P_1_	8
Partition coefficient P_2_	25
Mass transfer coefficient K_1_	0.08 cm/h
Mass transfer coefficient K_2_	0.04 cm/h

**Table 3:** Estimated parameter values for simulations of diclofenac at 32 °C.

Starting Concentration C_0_	5.00 mg/mL
Experiment time	51.5 h
Skin thickness	0.1 cm
Diffusion coefficient	1.8 × 10^−3^ cm^2^/h
Partition coefficient P_1_	5
Partition coefficient P_2_	10
Mass transfer coefficient K_1_	0.12 cm/h
Mass transfer coefficient K_2_	0.10 cm/h

**Table 4:** Estimated parameter values for simulations of caffeine at 32 °C.

Starting Concentration C_0_	5.00 mg/mL
Experiment time	53 h
Skin thickness	0.1 cm
Diffusion coefficient	5.0 × 10^−3^ cm^2^/h
Partition coefficient P_1_	8
Partition coefficient P_2_	23
Mass transfer coefficient K_1_	0.11 cm/h
Mass transfer coefficient K_2_	0.08 cm/h

## Data Availability

The data presented in this study are available on request from the corresponding author. The data are not publicly available at this time as it will be used in other ongoing studies.
